# Effect of an integrated control strategy for schistosomiasis japonica in the lower reaches of the Yangtze River, China: an evaluation from 2005 to 2008

**DOI:** 10.1186/1756-3305-4-243

**Published:** 2011-12-30

**Authors:** Le-Ping Sun, Wei Wang, You-Sheng Liang, Zeng-Xi Tian, Qing-Biao Hong, Kun Yang, Guo-Jing Yang, Jian-Rong Dai, Yang Gao

**Affiliations:** 1 Jiangsu Institute of Parasitic Diseases, 117 Yangxiang, Meiyuan, Wuxi 214064, Jiangsu Province, People's Republic of China; 2 Jiangsu Provincial Department of Health, 42 Zhongyang Road, Nanjing 210008, Jiangsu Province, People's Republic of China; 3 Yangzhou Municipal Center for Disease Control and Prevention, 35 Yanfu East Road, Yangzhou 225002, Jiangsu Province, People's Republic of China

**Keywords:** Schistosomiasis, *Schistosoma japonicum*, *Oncomelania hupensis*, Integrated control, Effect evaluation, The Yangtze River

## Abstract

**Background:**

Schistosomiasis japonica remains a major public health concern in China. There are many interventions implemented to control the transmission of the disease. The purpose of the present study was to investigate the effects of an integrated control strategy for schistosomiasis control.

**Methods:**

An integrated control strategy for schistosomiasis japonica with emphasis on removing cattle from snail-infested grasslands, providing farmers with mechanized farm equipment, improving sanitation by supplying tap water and building lavatories and latrines and providing boats with fecal-matter containers was implemented in 107 villages of the lower reaches of the Yangtze River, Jiangsu Province, China, during a 32-month period from May 2005 to 2008, and the effectiveness was investigated.

**Results:**

Following the effects of the comprehensive control, the snail habitat, infected snail habitat, snail infection rate, and *S. japonicum *prevalence in both humans and livestock all appeared a declining trend year by year, with reductions of 47.88%, 94.29%, 92.55%, 96.94%, and 100% compared with those before the comprehensive control. In addition, all of the 17 counties achieved the infection control in 2007, and 7 reached the criteria of transmission control in 2008. The confirmed snail habitats reduced from 107 to 20, and the acute infections have also been controlled for 2 successive years since 2007.

**Conclusions:**

The integrated control strategy for schistosomiasis japonica is effective to control the transmission of *S. japonicum*.

## Background

Human infection by the blood-fluke *Schistosoma japonicum *(Platyhelminthes: Trematoda) remains a major public health concern in the People's Republic of China, the Philippines, and parts of Indonesia [1-6]. In China, concerted control effects since the 1950s have dramatically reduced the number of the areas endemic for the parasite as well as the burden of disease among humans [7-10]. Nevertheless, in the remaining core endemic regions, mainly located along the middle and lower reaches of the Yangtze River and some mountainous areas of provinces of Yunnan and Sichuan, over 0.7 million people are estimated to be infected, with a further 30 million at risk of infection [11]. And currently in China, more than 80% of all human *S. japonicum *infections are concentrated in the marshland and lake regions of Jiangsu, Jiangxi, Anhui, Hunan and Hubei provinces where the interruption of transmission has been proved particularly difficult to be achieved [12,13].

Jiangsu province is located in the lower reaches of the Yangtze River in the east of China. Following the effect of flood of the upper reaches of the Yangtze River during the annual monsoon season [14,15], the marshlands along the Yangtze River operate in a "winter-land, summer-water" cycle, and vast grass-covered marshlands emerge after floodwaters recede, resulting in ideal breeding sites for *Oncomelania hupensis *survival and reproduction [16-18]. Historically, Jiangsu province suffered from a high prevalence of schistosomiasis japonica. After more than two decades of active comprehensive control with an emphasis on snail control by means of environmental improvement and mollusciciding, the province achieved the transmission control of the disease in 1976 [9,10,19]. Since the middle 1980s, the global strategy of schistosomiasis control has shifted from transmission control to morbidity control [20], following the development of the highly effective and safe schistosomicidal agent praziquantel [21,22]. In Jiangsu province, the praziquantel-based control strategy has been implemented on a large scale in all endemic areas since the late 1980s [23], particularly during the period of World Bank Loan Project for Schistosomiasis Control [24], which resulted in reduced morbidity caused by the parasite [25]. However, following the termination of the World Bank Loan Project for Schistosomiasis Control and the repeated flooding by the Yangtze River in the 1990s [15,16,19,26,27], acute human schistosomiasis cases were detected again and the area inhabited by infected *O. hupensis *snails started to increase [25,28]. Furthermore, surveillance studies suggest that chemotherapy-based programs, even those in combination with large-scale mollusciciding against snails, are unlikely to have much further impact upon prevalence levels among humans [11,12].

In 2005, the Jiangsu province proposed two goals for schistosomiasis control. First, by 2007, all of the 22 counties that were endemic for *S. japonicum *reached the criteria for infection control, and more than 50% of the counties reached the criteria for transmission control or transmission interruption. Second, by 2010, all of the schistosome-endemic counties reached the criteria for transmission control [29,30]. To achieve these two targets, since May, 2005, the Jiangsu province, according to the current local schistosomiasis epidemics, implemented an integrated control strategy for schistosomiasis japonica. Particularly emphasized were removing cattle from snail-infested grasslands, providing farmers with mechanized farm equipment, improving sanitation by supplying tap water and building lavatories and latrines, providing boats with fecal-matter containers, and other routine interventions like health education, snail control, and praziquantel-based synchronous chemotherapy for both infected humans and livestock [31]. In the present study, we describe the implementation of the integrated strategy and investigate the effectiveness of the strategy adopted in 107 villages from 17 counties along the lower reaches of the Yangtze River, Jiangsu province, during a 32-month period from May, 2005 to December, 2008.

## Methods

### Study area

**Figure 1:**
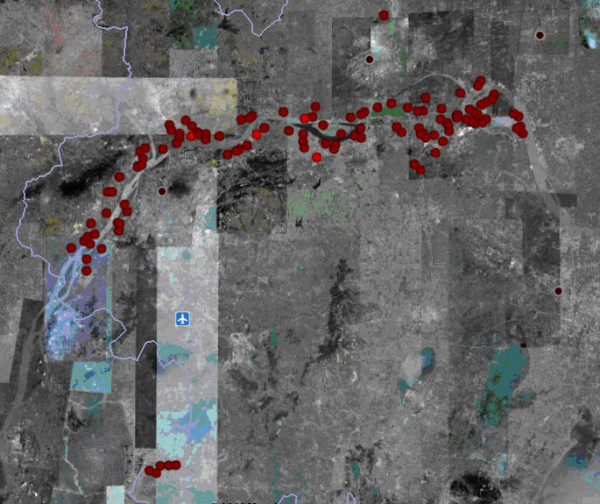
**Location of the study villages in the lower reaches of the Yangtze River, Jiangsu province, China**.

A total of 107 villages from 17 counties of Jiangsu province were included in the current study (Figure 1), where *S. japonicum*-infected snails were detected during the period between March, 2003 and April, 2005. The study areas had a total of 273,533 residents, 1323 bovine (including 971 cattle), 4379 sheep, 142 sluices and 103 river courses. Currently, 319 cases with advanced schistosomiasis were found and 977 cases with chronic schistosomiasis were identified from 2001 to 2005 [32]. From 2003 to April, 2005, a total of 266 sites infested with infected snails were detected in the study areas, with areas of 2877.85 hm^2^.

### Interventions to control sources of *S. japonicum *infection

During the study period from May, 2005 through 2008, considering that cattle were identified as the primary source of *S. japonicum *[33,34], all the 971 cattle were replaced with small farm machines to eliminate cattle as a source of infection to snails. And 8554 domestic animals (including bovine, sheep, pigs, dogs) were raised in pens to reduce or avoid the contamination of the grassland. To reduce humans as a source of infection in snails, the following interventions were implemented to attempt to reduce the transmission. A total of 725 households were supplied with tap water, 1907 fecal-matter container were supplied to the mobile boat fishermen and 75129 public latrines with three-cell septic tanks were constructed, so that human feces could be disposed of on land instead of directly into the lake.

### Comprehensive control of snail habitats

A comprehensive approach was employed to control snails by mollusciciding together with environmental modification. Over the 32-month study period, a total of 12671.52 hm^2 ^of snail habitats were treated with molluscicides like niclosamide. Environmental improvement such as constructing fish ponds, digging new ditches, building fruit trees and filling of infested areas was carried out by health sections, together with water resources development and agricultural and forestry projects. During the study period, 4173.55 hm^2 ^of snail habitats underwent environmental modification, 44 sluices were re-built for prevention of snail spread, and 217.4 km long river banks were hardened using concrete.

### Other routine control interventions

During the study period in all villages, some routine control activities were undertaken to control *S. japonicum *infection. These interventions included synchronous chemotherapy for both infected humans and livestock, and health education focusing on avoidance of snail-infested areas and associated lake water. From 2005 to 2008, 0.21 million people including 3922 boat fishermen were examined for schistosomiasis infections using serological screening with dipstick dye immunoassay (DDIA) [35-37], followed by the miracidium hatching test [32], 7264 high-risk populations were treated with praziquantel at a single oral dose of 40 mg/kg for expanded chemotherapy, and 0.55 million persons received health education by means of cartoons, videotapes, comic-style booklets, billboards, sessions and other media.

### Infection in humans and livestock

During the period of schistosomiasis non-transmission of each year, more than 90% of individuals aged 6-60 years in each study village were screened for *S. japonicum *antibodies using the DDIA technique [35-37], and the miracidium hatching test was employed in those seropositive individuals for definitive diagnosis of infections [32]. The miracidium hatching test was used for detecting infection of *S. japonicum *in livestock [32]. The seroprevalence and parasitological prevalence of *S. japonicum *were recorded and calculated.

### Effect evaluation of snail control

From 2005 to 2008, once-yearly (from April to May) a snail survey was carried out by means of a systematic sampling technique along the river banks and in marshland and ditches around the study villages [32]. A snail collection device made of iron wire and consisting of a 0.1 m^2 ^square frame was placed every 20 m along the survey line. All snails within the frame were collected, enumerated, crushed and examined for *S. japonicum *infection using a microscopy. Various indices were recorded, namely snail habitats, density of living snails, density of infected snails and the snail infection rate.

### Ethical approval

This study was approved by the Ethics Review Committee of Jiangsu Province, Jiangsu Institute of Parasitic Diseases, and National Institute of Parasitic Diseases, Chinese Center for Disease Control and Prevention.

### Statistical analysis

All data were entered in Excel (Microsoft Corporation; Redmond, WA, USA) and all statistical analyses were performed using the statistical software Statistical Package for the Social Sciences Version 13.0 (SPSS Inc., Chicago, IL, USA). Differences of proportions were tested for statistical significance with the chi-square test. A *P*-value < 0.05 was considered significant.

## Results

### *S. japonicum *infections in humans and livestock

**Figure 2:**
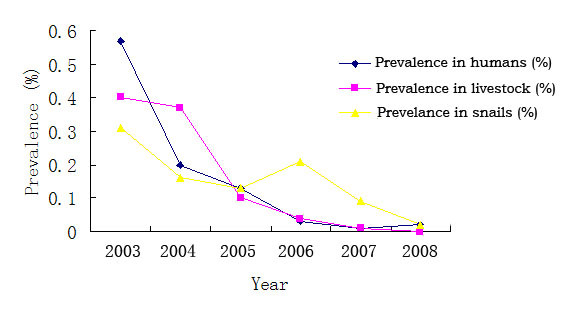
**Evolution of average *S. japonicum *prevalence in humans, livestock and *O. hupensis *snails in the study villages from 2003 to 2008**.

The prevalences of *S. japonicum *were 0.57%, 0.2%, 0.13%, 0.03%, 0.01% and 0.02%, respectively in humans, and 0.4%, 0.37%, 0.1%, 0.04%, 0.01% and 0 in livestock from 2003 to 2008 (Table 1, Figure 2). Between 2003 and 2008, the annual acute infections were 116, 38, 11, 2, 0 and 0, presenting a significant declining trend. After the implementation of the integrated schistosomiasis control strategy, the seroprevalence, parasitological prevalence and prevalence of *S. japonicum *in humans reduced by 81.17%, 90.35% and 96.94%, respectively, and 100% of reductions in prevalence of livestock and bovine and acute infections were also achieved (Table 2), in comparison with those rates before the implementation of the integrated strategy (all *P *values < 0.01).

**Table 1:** *S. japonicum *prevalence in humans and livestock and acute infections in the 107 villages in a pilot control program of Jiangsu Province, from 2003 to 2008

Year	No. people examined	No. infected people	Prevalence of residents (%)	No. acute infections	No. livestock detected		No. infected livestock		Prevalence of livestock (%)	
					
					Total	In: bovine detected	Total	Infected bovine	Total	In: bovine
2003	140868	802	0.57	116	9849	5424	39	35	0.4	0.65
2004	237443	478	0.2	38	9526	4956	35	15	0.37	0.3
2005	229979	298	0.13	11	15928	3900	16	8	0.1	0.21
2006	264357	90	0.03	2	16170	2724	6	3	0.04	0.11
2007	305719	45	0.01	0	14215	2198	2	2	0.01	0.09
2008	252323	44	0.02	0	13705	1604	0	0	0	0

**Table 2:** Comparison of the control effects before and after the implementation of the integrated schistosomiasis control strategy in 107 villages of Jiangsu Province

Indicator	Before the implementation of the integrated control strategy	After the implementation of the integrated control strategy	Reductions (%)
Snail habitats (hm^2^)	7243.03	3775.26	47.88
Infected snail habitats (hm^2^)	2173.93	124.19	94.29
No. villages with infected snails	80	20	75
Seroprevalence in humans (%)	4.5	0.85	81.17
Parasitological prevalence in humans (%)	0.06	0.01	90.35
Prevalence in humans (%)	0.57	0.02	96.94
Acute infections	116	0	100
Prevalence in livestock (%)	0.4	0	100
Prevalence in bovine (%)	0.65	0	100
Snail infections (%)	0.31	0.02	92.55

### *S. japonicum *infections in snails

From 2003 to 2008, the confirmed snail habitats out of all field survey sites were 80, 90, 60, 44, 41 and 20, respectively. The total area of snail habitats was reduced from 7243.06 hm^2 ^to 3775.26 hm^2^, and the area where infected snails were detected decreased from 2173.93 hm^2 ^to 124.19 hm^2 ^over the study period. The prevalences of *S. japonicum *infections in the snails collected from 2003 to 2008 were 0.31%, 0.16%, 0.l3%, 0.21%, 0.09% and 0.02%, respectively (Table 3). After the implementation of the integrated strategy, the snail host and *S. japonicum *infections in snails were controlled significantly, with reductions in snail habitat, infected snail habitat and snail infections of 47.88%, 94.29% and 92.55%, respectively (Table 2).

**Table 3:** Changes of *Oncomelania hupensis *snail in the 107 villages in a pilot control program of Jiangsu Province, from 2003 to 2008

Year	Snail habitat (hm^2^)	Infected snail habitat (hm^2^)	No. snails dissected	No. infected snails	Infection rate of snails (%)	No. villages with infected snails
2003	7243.06	2173.93	585298	1808	0.31	80
2004	7625.23	2130.53	597783	950	0.16	90
2005	7701.07	1767.70	551843	705	0.13	60
2006	5887.96	1444.34	344905	735	0.21	44
2007	4487.75	802.73	296868	254	0.09	41
2008	3775.26	124.19	295384	68	0.02	20

Up to 2007, all of the 17 counties achieved the infection control. In 2008, 7 out of the 17 counties reached the criteria of transmission control, and *S. japonicum*-infected snails were eliminated in 60 out of the 117 study villages.

## Discussion

Historically, Jiangsu Province suffered from a very high prevalence of schistosomiasis japonica in China, 10 out of the 13 cities of the province were endemic for *S. japonicum*, with accumulated schistosomiasis cases of more than 2.5 million, and accumulated snail habitats of about 1.5 billion m^2 ^[7,9,10,38]. During the past 5 decades, the Jiangsu Provincial government placed a high priority on the control of schistosomiasis and had carried out many control programs. These effective interventions have resulted in substantial reductions in the prevalence of *S. japonicum *in humans and livestock, and snail and infected snail habitats [28,29]. The remaining core endemic areas were mainly located in the marshlands along the Yangtze River and those rivers that connect with the Yangtze River [39]. In terms of the present schistosomiasis epidemics, how to highlight the control emphasis and then implement the effective interventions is a key point to promote further control programs. In 2004, the Jiangsu Provincial government formulated the Mid- and Long-term Plan for Prevention and Control of Schistosomiasis in Jiangsu Province (2005-2010) based on control of schistosomiasis in Jiangsu section of the Yangtze River, and initiated an integrated control program for schistosomiasis in all villages that are endemic for *S. japonicum *since 2005 [31,40,41]. The strategy focused on 117 villages of the province where *S. japonicum*-infected snails were found. Through the field implementation of the integrated schistosomiasis control, significant control effects were achieved. In addition, the new strategy provided a novel pattern for control of schistosomiasis, namely integrating all kinds of resources and boosting the whole villages.

With the gradual decline of schistosomiasis, the Chinese government has implemented a stratified control pattern based on villages with different intensities of *S. japonicum *infection [33,34]. However, the stratification is mainly based on the infection rates of *S. japonicum *in both humans and livestock, which is increasingly meaningless in areas with low infection intensities [42,43]. Since 2005, we targeted the 107 villages of the province (belonging to 17 counties) where infected snails were detected between March, 2003 and April, 2005, and carried out a comprehensive control strategy for schistosomiasis japonica in the field for a successive four years. Following the effects of the comprehensive control, the snail habitat, infected snail habitat, snail infection rate, and *S. japonicum *prevalence in both humans and livestock all appeared a declining trend year by year, with reductions of 47.88%, 94.29%, 92.55%, 96.94%, and 100% compared with those before the comprehensive control. In addition, all of the 17 counties achieved the infection control in 2007, and 7 reached the criteria of transmission control in 2008. The confirmed snail habitats reduced from 107 to 20, and the acute infections have also been controlled for a successive 2 years since 2007. It is indicated that the integrated schistosomiasis control strategy with emphasis on elimination of the snail host could promote the control progress in those regions with low infection intensities.

Schistosomiasis control is a systematic project involving sectors of water conservancy, agriculture, forestry, health, etc [44]. The effects of combination of resources from different sectors on schistosomiasis control remains unclear, and the better utilization would be of great value for further promotion of the control achievements. The present study showed, under the application of the same mollusciciding, the snail habitats kept vast, the prevalence in humans and livestock reduced but still maintained in a high level, and the acute infections were not effectively controlled from 2003 to April, 2005. By contrast, since May, 2005, when the government initiated the integrated control program, the snail habitats reduced dramatically, the prevalence in humans, livestock and snails quickly decreased to a low level, and the acute infections were eliminated. In 2006, a higher snail infection rate was observed compared with those in 2004 and 2005, this was because mollusciciding was not effectively implemented in 1 of 107 villages, leading to a failure in the control of snail control. However, all of the other indicators like the snail habitat, infected snail habitat, number of villages with infected snails, reduced compared with those in both 2004 and 2005.

## Conclusions

The integrated control strategy for schistosomiasis japonica described here is effective to control the transmission of *S. japonicum*, and it established a new schistosomiasis control pattern that integrates multi-sector resources. Further studies should be carried out to investigate the effects of the strategy in other schistosome-endemic regions of China with comparable ecological, cultural and socio-economic characteristics.

## Competing interests

The authors declare that they have no competing interests.

## Authors' contributions

LPS and YSL conceived and designed the study. LPS collected the data. LPS, WW, ZXT, QBH, GJY, KY, JRD implemented the study. LPS and WW carried out the statistical analysis and prepared the manuscript. YSL revised and finalized the manuscript. All of the authors read and approved the final version of the manuscript.

## References

[B1] Engels D Chitsulo L Montresor A Savioli L The global epidemiological situation of schistosomiasis and new approaches to control and research Acta Trop 2002 82 139 146 10.1016/S0001-706X(02)00045-1 12020886 PMC5633073

[B2] Chitsulo L Loverde P Engels D Schistosomiasis Nat Rev Microbiol 2004 2 12 13 15035004 10.1038/nrmicro801

[B3] Steinmann P Keiser J Bos R Tanner M Utzinger J Schistosomiasis and water resources development: systematic review, meta-analysis, and estimates of people at risk Lancet Infect Dis 2006 6 411 425 10.1016/S1473-3099(06)70521-7 16790382

[B4] Utzinger J Bergquist R Olveda R Zhou XN Important helminth infections in Southeast Asia diversity, potential for control and prospects for elimination Adv Parasitol 2010 72 1 30 20624526 10.1016/S0065-308X(10)72001-7

[B5] Zhou XN Bergquist R Leonardo L Yang GJ Yang K Sudomo M Olveda R Schistosomiasis japonica control and research needs Adv Parasitol 2010 72 145 178 20624531 10.1016/S0065-308X(10)72006-6

[B6] Gryseels B Polman K Clerinx J Kestens L Human schistosomiasis Lancet 2006 368 1106 1118 10.1016/S0140-6736(06)69440-3 16997665

[B7] Yuan H Jiang Q Zhao G He N Achievements of schistosomiasis control in China Mem Inst Oswaldo Cruz 2002 97 187 189 12426618 10.1590/s0074-02762002000900036

[B8] Engels D Wang LY Palmer KL Control of schistosomiasis in China Acta Trop 2005 96 67 68 10.1016/j.actatropica.2005.07.004 16125656

[B9] Zhou XN Wang LY Chen MG Wu XH Jiang QW Chen XY Zheng J Utzinger J The public health significance and control of schistosomiasis in China--then and now Acta Trop 2005 96 97 105 10.1016/j.actatropica.2005.07.005 16125655

[B10] Wang LD Utzinger J Zhou XN Schistosomiasis control: experiences and lessons from China Lancet 2008 372 1793 1795 10.1016/S0140-6736(08)61358-6 18930529 PMC7135384

[B11] Zhou XN Guo JG Wu XH Jiang QW Zheng J Dang H Wang XH Xu J Zhu HQ Wu GL Li YS Xu XJ Chen HG Wang TP Zhu YC Qiu DC Dong XQ Zhao GM Zhang SJ Zhao NQ Xia G Wang LY Zhang SQ Lin DD Chen MG Hao Y Epidemiology of schistosomiasis in the People's Republic of China, 2004 Emerg Infect Dis 2007 13 1470 1476 18257989 10.3201/eid1310.061423 PMC2851518

[B12] Zhao GM Zhao Q Jiang QW Chen XY Wang LY Yuan HC Surveillance for schistosomiasis japonica in China from 2000 to 2003 Acta Trop 2005 96 288 295 10.1016/j.actatropica.2005.07.023 16202597

[B13] Li SZ Luz A Wang XH Xu LL Wang Q Qian YJ Wu XH Guo JG Xia G Wang LY Zhou XN Schistosomiasis in China: acute infections during 2005-2008 Chin Med J (Engl) 2009 122 1009 1014 19493433

[B14] Dudgeon D Arthington AH Gessner MO Kawabata Z-I Knowler DJ Lévêque C Naiman RJ Prieur-Richard A-H Soto D Stiassny MLJ Sullivan CA Freshwater biodiversity: importance, threats, status and conservation challenges Biol Rev 2005 81 163 182 16336747 10.1017/S1464793105006950

[B15] Wu XH Zhang SQ Xu XJ Huang YX Steinmann P Utzinger J Wang TP Xu J Zheng J Zhou XN Effect of floods on the transmission of schistosomiasis in the Yangtze River valley, People's Republic of China Parasitol Int 2008 57 271 276 10.1016/j.parint.2008.04.004 18499513

[B16] Huang YX Rong GR Cai G Gao ZH Zhou XN Zhu YC Effects of flood on snail distribution in marshland along Yangtze River in Jiangsu Province Chin J Schisto Control 2000 12 346 349

[B17] Seto EYW Wu WP Liu HY Chen HG Hubbard A Holt A Davis GM Impact of changing water levels and weather on *Oncomelania hupensis hupensis *populations, the snail host of *Schistosoma jaonicum*, downstream of the Three Gorges Dam Ecohealth 2008 5 149 158 10.1007/s10393-008-0169-x 18787918

[B18] Zhu HM Xiang S Yang K Wu XH Zhou XN Three Gorges Dam and its impact on the potential transmission of schistosomiasis in regions along the Yangtze River Ecohealth 2008 5 137 148 10.1007/s10393-008-0168-y 18787917

[B19] Huang YX Li B Wang LP Zhu YC Zhou XN Zhao YJ Evaluation on the disease control effect of World Bank Loan Schistosomiasis Control Project in Jiangsu Province Chin J Schisto Control 2001 13 292 295 (in Chinese)

[B20] WHO The Control of schistosomiasis. Second Report of the WHO Expert Committee WHO Tech Rep Ser 1993 World Health Organziation, Geneva No. 830 8322462

[B21] Gönnert R Andrews P Praziquantel, a new broad-spectrum antischistosomal agent Z Parasitenkd 1977 52 129 150 10.1007/BF00389899 410178

[B22] Seubert J Pohlke R Loebich F Synthesis and properties of praziquantel, a novel broad spectrum anthelmintic with excellent activity against schistosomes and cestodes Experientia 1977 33 1036 1037 10.1007/BF01945954 891804

[B23] Jiang QW Wang LY Guo JG Chen MG Zhou XN Engels D Morbidity control of schistosomiasis in China Acta Trop 2002 82 115 125 10.1016/S0001-706X(02)00006-2 12020884

[B24] Yuan H Jiagang G Bergquist R Tanner M Xianyi C Huanzeng W The 1992-1999 World Bank Schistosomiasis Research Initiative in China: outcome and perspectives Parasitol Int 2000 49 195 207 10.1016/S1383-5769(00)00045-3 11426575

[B25] Hong QB Sun LP Huang YX Li W Yang K Gao Y Zhang LH Gao Y Zeng YL Zhou M Liang YS Zhu YC Cai G The third sampling survey of schistosomiasis in Jiangsu Province Chin J Schisto Control 2005 17 268 272 (in Chinese)

[B26] Huang YX Sun LP Hong QB Gao Y Zhang LH Gao Y Chen H Guo JH Liang YS Zhu YC Longitudinal observation on fluctuation trend of distribution and spread of *Oncomelania *snails after flood water in marshland of lower reaches of Yangtze River Chin J Schisto Control 2004 16 253 256 (in Chinese)

[B27] Chen XY Wang LY Cai JM Zhou XN Zheng J Guo JG Wu XH Engels D Chen MG Schistosomiasis control in China: the impact of a 10-year World Bank Loan Project (1992-2001) Bull World Health Organ 2005 83 43 48 15682248 PMC2623468

[B28] Hong QB Huang YX Cai G Sun LP Gao Y Zhang LH Gao Y Liang YS Surveillance on schistosomiasis in Jiangsu Province from 2000 to 2004 J Path Biol 2007 2 213 126 (in Chinese)

[B29] Huang YX Hong QB Sun LP Hang DR Li W Zhang JF Liang YS Mid-term effectiveness of medium-and-long-term programme of prevention and control of schistosomiasis in Jiangsu Province Chin J Schisto Control 2008 20 245 250

[B30] Sun LP Liang YS Wu HH Tian ZX Dai JR Yang K Hong QB Zhou XN Yang GJ A Google Earth-based surveillance system for schistosomiasis japonica implemented in the lower reaches of the Yangtze River, China Parasit Vectors 2011 4 223 10.1186/1756-3305-4-223 22117601 PMC3250965

[B31] Wang LD Chen HG Guo JG Zeng XJ Hong XL Xiong JJ Wu XH Wang XH Wang LY Xia G Hao Y Chin DP Zhou XN A strategy to control transmission of *Schistosoma japonicum *in China N Engl J Med 2009 360 121 128 10.1056/NEJMoa0800135 19129526

[B32] MOH Handbook for Schistosomiasis Control 2000 Shanghai: Shanghai Science & Technology Press (in Chinese)

[B33] Wang LD Key point of schistosomiasis control in China lies in stool management from humans and animals Chin J Epidemiol 2005 26 929 930

[B34] Wang TP Vang Johansen M Zhang SQ Wang FF Wu WD Zhang GH Pan XP Ju Y Ombjerg N Transmission control of *Schistosoma japonicum *by humans and domestic animals in the Yangtze River valley, Anhui Province, China Acta Trop 2005 96 198 204 10.1016/j.actatropica.2005.07.017 16188215

[B35] Zhu Y He W Liang Y Xu M Yu C Hua W Chao G Development of a rapid, simple dipstick dye immunoassay for schistosomiasis diagnosis J Immunol Methods 2002 266 1 5 10.1016/S0022-1759(02)00086-8 12133617

[B36] Zhu Y Socheat D Bounlu K Liang YS Sinuon M Insisiengmay S He W Xu M Shi WZ Bergquist R Application of dipstick dye immunoassay (DDIA) kit for the diagnosis of schistosomiasis mekongi Acta Trop 2005 96 137 141 10.1016/j.actatropica.2005.07.008 16143289

[B37] Xu J Feng T Lin DD Wang QZ Tang L Wu XH Guo JG Peeling RW Zhou XN Performance of a dipstick dye immunoassay for rapid screening of Schistosoma japonicum infection in areas with low endemicity Parasit Vectors 2011 4 87 10.1186/1756-3305-4-87 21599944 PMC3123290

[B38] Utzinger J Zhou XN Chen MG Bergquist R Conquering schistosomiasis in China: the long march Acta Trop 2005 96 69 96 16312039 10.1016/j.actatropica.2005.08.004

[B39] Huang YX Cai G Wu F Pao H Song HT Zhang XB Cao SL Zhu YC Survey of present situation of marshland and snail habitat areas and study on control strategy of marshland *Oncomelania hupensis *in 5 cities along the Yangtze River in Jiangsu Province Chin J Schisto Control 2000 12 86 90 (in Chinese)

[B40] Wang LD Guo JG Wu XH Chen HG Wang TP Zhu SP Zhang ZH Steinmann P Yang GJ Wang SP Wu ZD Wang LY Hao Y Bergquist R Utzinger J Zhou XN China's new strategy to block Schistosoma japonicum transmission: experiences and impact beyond schistosomiasis Trop Med Int Health 2009 14 1475 1483 10.1111/j.1365-3156.2009.02403.x 19793080

[B41] Sun LP Liang YS Wu HH Tian ZX Hong QB Huang YX Gao Y Mie J Gao Y Xie CY Zhang LH Wu F Hu XS Effect evaluation of comprehensive control for schistosomiasis in key villages of Jiangsu Province Chin J Schisto Control 2009 21 285 289 (in Chinese)

[B42] Zhou XN Jiang QW Sun LP Wang TP Hong QB Zhao GM Wen LY Yin ZC Wu XH Lin DD Schistosomiasis control and surveillance in China Chin J Schisto Control 2005 17 161 165 (in Chinese)

[B43] Zhou XN Jia TW Guo JG Wang LY Jiang QW Management model and its evolution in schistosomiasis control programme of China Chin J Schisto Control 2010 22 1 4 (in Chinese)

[B44] Yuan H Achievement and experiences of schistosomiasis control in China Chin J Epidemiol 1999 2 3 6 (in Chinese)

